# Supplementary material for: A concerted increase in readthrough and intron retention drives transposon expression during aging and senescence
Source: eLife. 2024 Apr 3;12:RP87811. doi: 10.7554/eLife.87811 (PMC10990488; doi:10.7554/eLife.87811)
Supplement: Supplementary file 1. [file elife-87811-supp1.docx]

| **Dataset** | **Transp. family** | **up** | **down** | **Fraction up** | **mean log2 fc** |
| --- | --- | --- | --- | --- | --- |
| Aging | LTR | 770 | 163 | 0.825 | 0.328 |
|  | DNA | 845 | 70 | 0.923 | 0.430 |
|  | LINE | 3337 | 352 | 0.905 | 0.408 |
|  | SINE | 1518 | 589 | 0.720 | 0.326 |
|  |  |  |  |  |  |
|  | **Transp. family** | **up** | **down** | **Fraction up** | **mean log2 fc** |
| Senescence | LTR | 1640 | 183 | 0.900 | 2.445 |
|  | DNA | 611 | 149 | 0.804 | 1.766 |
|  | LINE | 2901 | 481 | 0.858 | 2.097 |
|  | SINE | 1960 | 596 | 0.767 | 1.581 |

**Supplementary File 1a**Numbers of significantly up- and downregulated transposon families with aging and cellular senescence. Mean log2-fold change (“log2 fc”) and fraction upregulated is also shown. Aging data from Fleischer et al. (2018) and senescence data from Colombo et al. (2018).

| **Aging** | sig. readthrough | no sig. readthrough | Fraction |  |
| --- | --- | --- | --- | --- |
| transposon | 579 | 63203 | 0.009 |  |
| sig. transposon | 158 | 7535 | 0.021 | p<0.0001 |
| **Senescence** |  |  |  |  |
| transposon | 1840 | 122333 | 0.015 |  |
| sig. transposon | 574 | 8156 | 0.070 | p<0.0001 |
|  |  |  |  |  |
| **Aging** | any. readthrough | no readthrough | Fraction |  |
| transposon | 1357 | 62425 | 0.022 |  |
| sig. transposon | 265 | 7428 | 0.036 | p<0.0001 |
| **Senescence** |  |  |  |  |
| transposon | 6061 | 118112 | 0.051 |  |
| sig. transposon | 751 | 7979 | 0.094 | p<0.0001 |

**Supplementary File 1b**Number of transposons and significant transposons that are located in readthrough regions. p-value by Fisher’s exact test.

| **Dataset** | **Analysis** | **Transposons** | **R** | **n** | **P-value** |
| --- | --- | --- | --- | --- | --- |
| Aging  Fleischer et al. | sig vs sig | downstream | 0.33 | 156 | p<0.0001 |
|  | sig vs sig | any | 0.11 | 465 | p=0.022 |
|  | all vs all | downstream | 0.29 | 1337 | p<0.0001 |
|  | all vs all | all | 0.13 | 9418 | p<0.0001 |
|  |  |  |  |  |  |
| Senescence  Colombo et al. | sig vs sig | downstream | 0.88 | 562 | p<0.0001 |
|  | sig vs sig | any | 0.74 | 1246 | p<0.0001 |
|  | all vs all | downstream | 0.76 | 5971 | p<0.0001 |
|  | all vs all | all | 0.45 | 33232 | p<0.0001 |

**Supplementary File 1c**Correlation (R) between the expression levels of transposons and readthrough at the adjacent gene. We included either all significant elements in this analysis (“sig vs sig”) or all expressed elements (“all vs all”). Transposons in the “any” category can be located upstream, intragenic, intronic or downstream to the gene and its readthrough region.

| **Model** | **Adjusted R-squared** |
| --- | --- |
| transposon ~ intron | 0.405 |
| transposon ~ intron + readthrough | 0.562 |

**Supplementary File 1d**Comparison of two linear models to predict normalized transposon expression in the aging dataset using either normalized intron retention levels or also including normalized readthrough levels. P<0.0001 by ANOVA.

| **Senescence** | LINE1 | non LINE1 | | **Aging** | LINE1 | non LINE1 |
| --- | --- | --- | --- | --- | --- | --- |
| intragenic | 63.9 | 59.0 |  | intragenic | 80.4 | 87.6 |
| intronic | 53.4 | 46.2 |  | intronic | 52.1 | 38.2 |
| downstream | 13.1 | 20.4 |  | downstream | 15.3 | 9.0 |
| upstream | 12.2 | 12.0 |  | upstream | 3.6 | 3.1 |
| sum | 89.2 | 91.4 |  | sum | 99.3 | 99.6 |

**Supplementary File 1e**Percentage of transposons that are intragenic, intronic, downstream or upstream of genes (within 25kb) and the sum total of intragenic, downstream and upstream.

| **Transposon class** | **Aging** | **Senescence** |
| --- | --- | --- |
| active, significant LINE-1 | 0.65 | 0.97 |
| significant LINE-1 | 0.94 | 0.88 |
| significant transposon | 0.84 | 0.84 |
| any transposon | 0.61 | 0.62 |

**Supplementary File 1f**The fraction of transposons upregulated with aging or cellular senescence in each class.

| **Dataset** | **Group** | **Transposons** | **any L1** | **L1 w/**  **ORF1p** | **P-value** | **L1 w/ ORF2p** | **P-value** |
| --- | --- | --- | --- | --- | --- | --- | --- |
| Aging  Fleischer et al. | outliers | 283 | 91 | 0 | NS | 0 | NA |
|  | sig | 7693 | 2820 | 8 | <0.05 | 0 | NA |
|  | all | 63782 | 12214 | 30 | NA | 2 | NA |
|  |  |  |  |  |  |  |  |
| Senescence  Colombo et al. | outliers | 265 | 86 | 2 | <0.05 | 0 | NA |
|  | sig | 8730 | 2506 | 19 | <0.0001 | 2 | NS |
|  | all | 124173 | 27167 | 95 | NA | 24 | NA |

**Supplementary File 1g**Numbers of ORF1p- and ORF2p-encoding LINE-1 elements among all expressed transposons, transposons significantly changed with aging and transposons with higher-than-expected expression during aging (“outliers”). The p-value was determined by comparison with randomly sampled elements from the “all” group.
